# Supplementary material for: A CandiChrome toolkit for multicolor labeling of Candida cells
Source: Sci Rep. 2026 Aug 11;16:37490. doi: 10.1038/s41598-026-64709-z (PMC13747812; doi:10.1038/s41598-026-64709-z)
Supplement: Supplementary file 1 [file 41598_2026_64709_MOESM1_ESM.pdf]

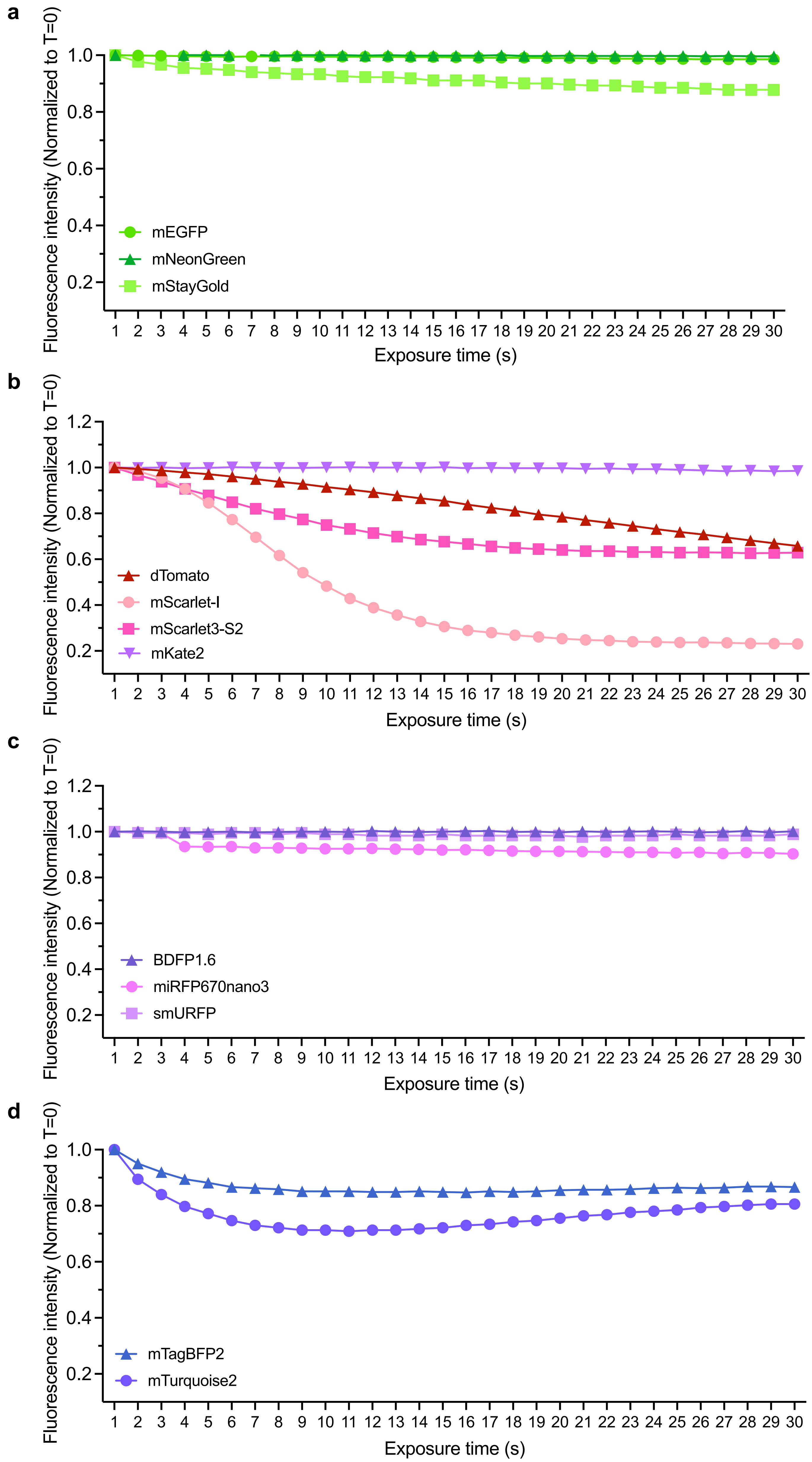

Figure S1. Extended photostability analysis of candidate CandiChrome fluorophores over 30 consecutive exposures.
To assess fluorophore durability under longer repeated-imaging conditions, cells were grown overnight in SCD at 30°C and fluorescence intensity was measured during 30 consecutive 1-s exposures using 30% light intensity under the same illumination settings as the initial photobleaching analysis. Signal intensity was normalized to the first exposure for each fluorophore. **a,** Green fluorescent proteins: mEGFP, mNeonGreen, and mStayGold. **b,** Red fluorescent proteins: dTomato, mScarlet-I, mScarlet3-S2, and mKate2. **c,** Far-red fluorescent proteins: BDFP1.6, miRFP670nano3, and smURFP. **d,** Blue/cyan fluorescent proteins: mTagBFP2 and mTurquoise2.

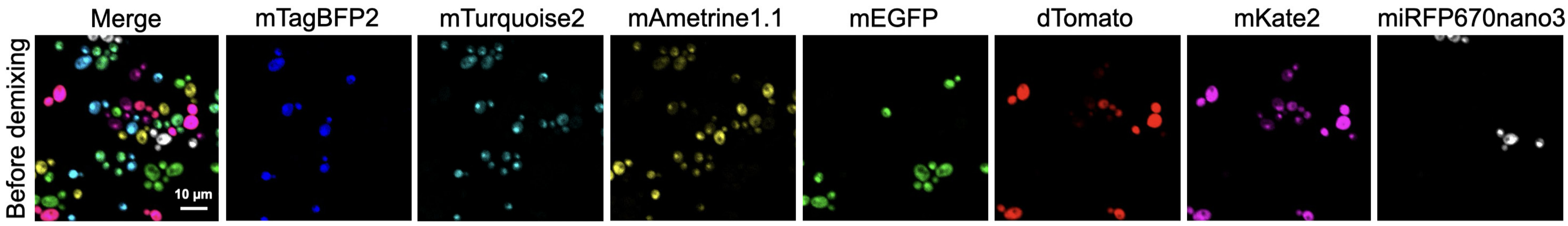

Ex. (nm)	405	405	405	488	561	561	640
Em. (nm)	430-460	465-505	515-550	515-550	510-550	610-650	610-650
mTagBFP2	100.0	305.0	76.7	0.0	0.3	0.6	0.0
mTurquoise2	22.2	100.0	39.0	0.4	0.8	2.0	3.2
mAmetrine1.1	0.1	2.0	100.0	0.0	0.0	0.0	0.0
mEGFP	0.2	1.7	3.5	100.0	0.0	0.0	0.0
dTomato	0.7	5.7	2.0	0.2	100.0	177.4	0.1
mKate2	0.3	2.1	1.1	0.0	9.9	100.0	0.6
miRFP670nano3	0.0	0.3	0.1	0.0	0.0	0.0	100.0

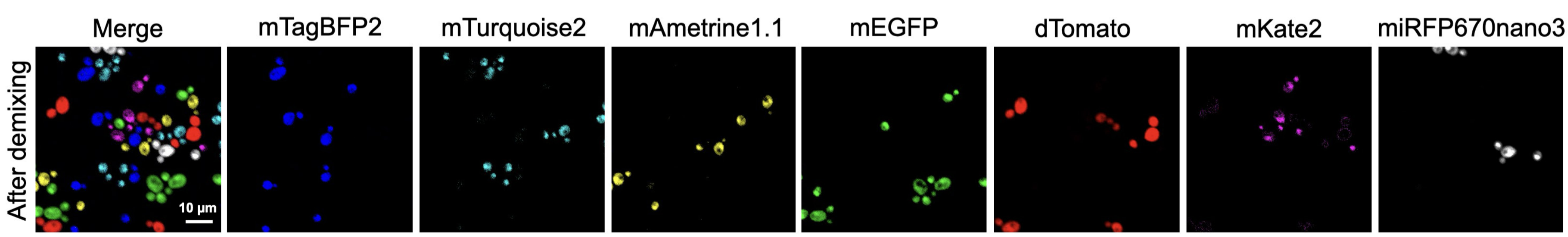

Ex. (nm)	405	405	405	488	561	561	640
Em. (nm)	430-460	465-505	515-550	515-550	510-550	610-650	610-650
mTagBFP2	100.0	7.1	0.6	0.0	0.1	0.0	0.0
mTurquoise2	2.5	100.0	0.6	0.0	0.0	0.0	0.0
mAmetrine1.1	0.4	0.0	100.0	0.0	0.0	0.0	0.0
mEGFP	0.5	0.0	0.0	100.0	0.0	0.0	0.0
dTomato	1.7	0.0	0.0	0.1	100.0	8.1	0.1
mKate2	0.8	0.3	0.0	0.0	1.6	100.0	0.3
miRFP670nano3	0.2	0.0	0.0	0.0	0.0	0.0	100.0

Figure S2. Spectral demixing and channel spillover analysis of the CandiChrome microscopy panel using an Evident FV3000 confocal microscope.

Representative fluorescence images of mixed *C. albicans* populations expressing the indicated CandiChrome fluorophores before and after spectral demixing. Cells were grown overnight in SCD at 30 °C and imaged on an FV3000 confocal microscope using the indicated excitation lasers and emission detection windows. Spectral demixing was performed using the instrument's built-in software. The matrices show relative fluorescence signal across each detection channel before and after demixing, normalized to the expected channel for each fluorophore. Scale bar, 10 µm.

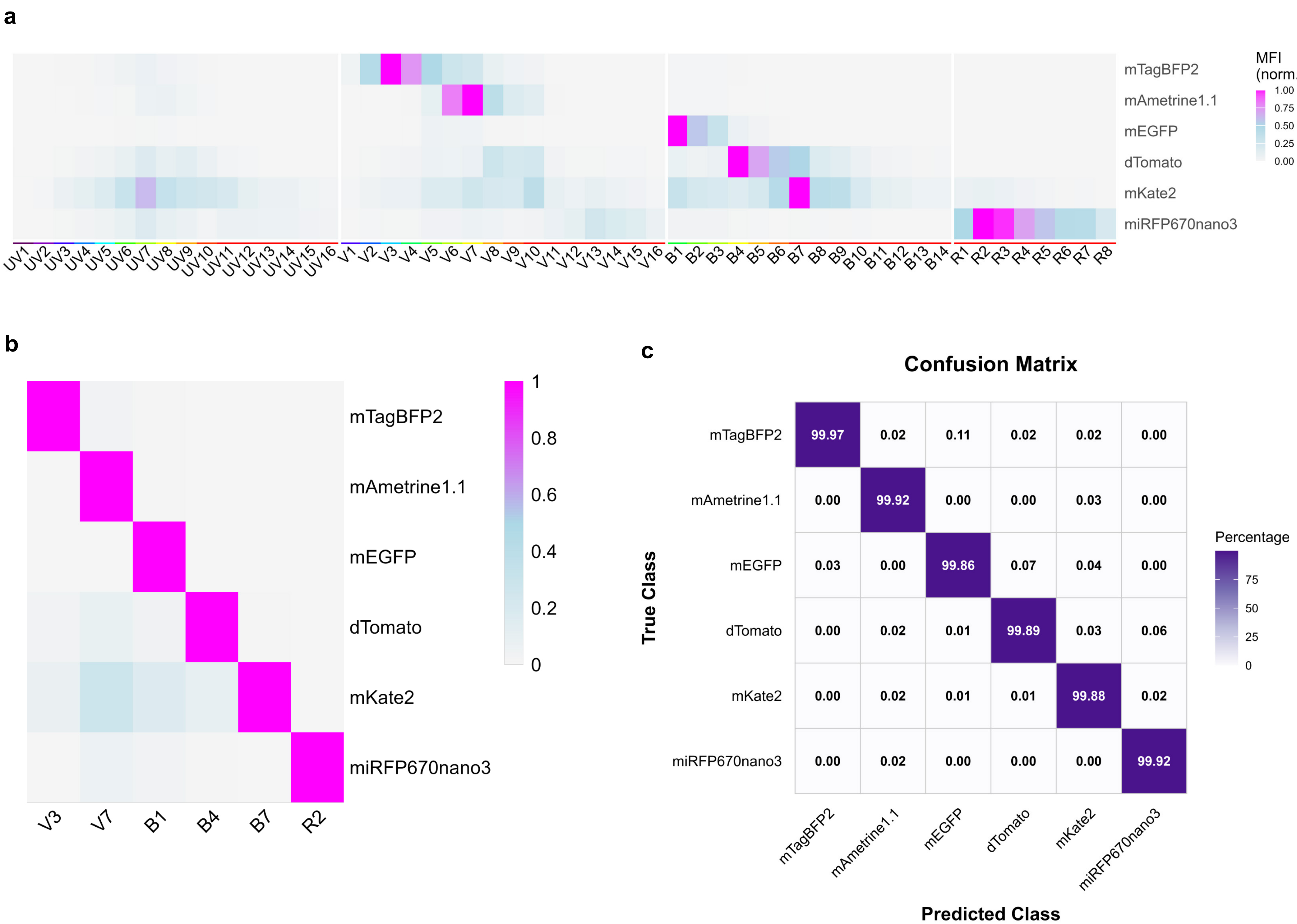

Figure S3. Spectral overlap and unmixing analysis of the six-color CandiChrome flow cytometry panel using an Aurora spectral flow cytometer.

Single-color labeled *C. albicans* strains expressing mTagBFP2, mAmetrine1.1, mEGFP, dTomato, mKate2, or miRFP670nano3 were grown overnight in SCD at 30 °C and analyzed on an Aurora spectral flow cytometer. **a,** Heatmap showing normalized median fluorescence intensity (MFI) from each single-color strain across all emission detection channels before spectral unmixing, illustrating the raw detector signal profiles and spectral overlap between fluorophores. **b,** Heatmap showing the corresponding unmixed signal matrix after spectral unmixing using the instrument's built-in software. **c,** Confusion matrix generated from the same single-color control data, showing the percentage of cells from each true fluorescent population assigned to each predicted fluorescent population after spectral unmixing.

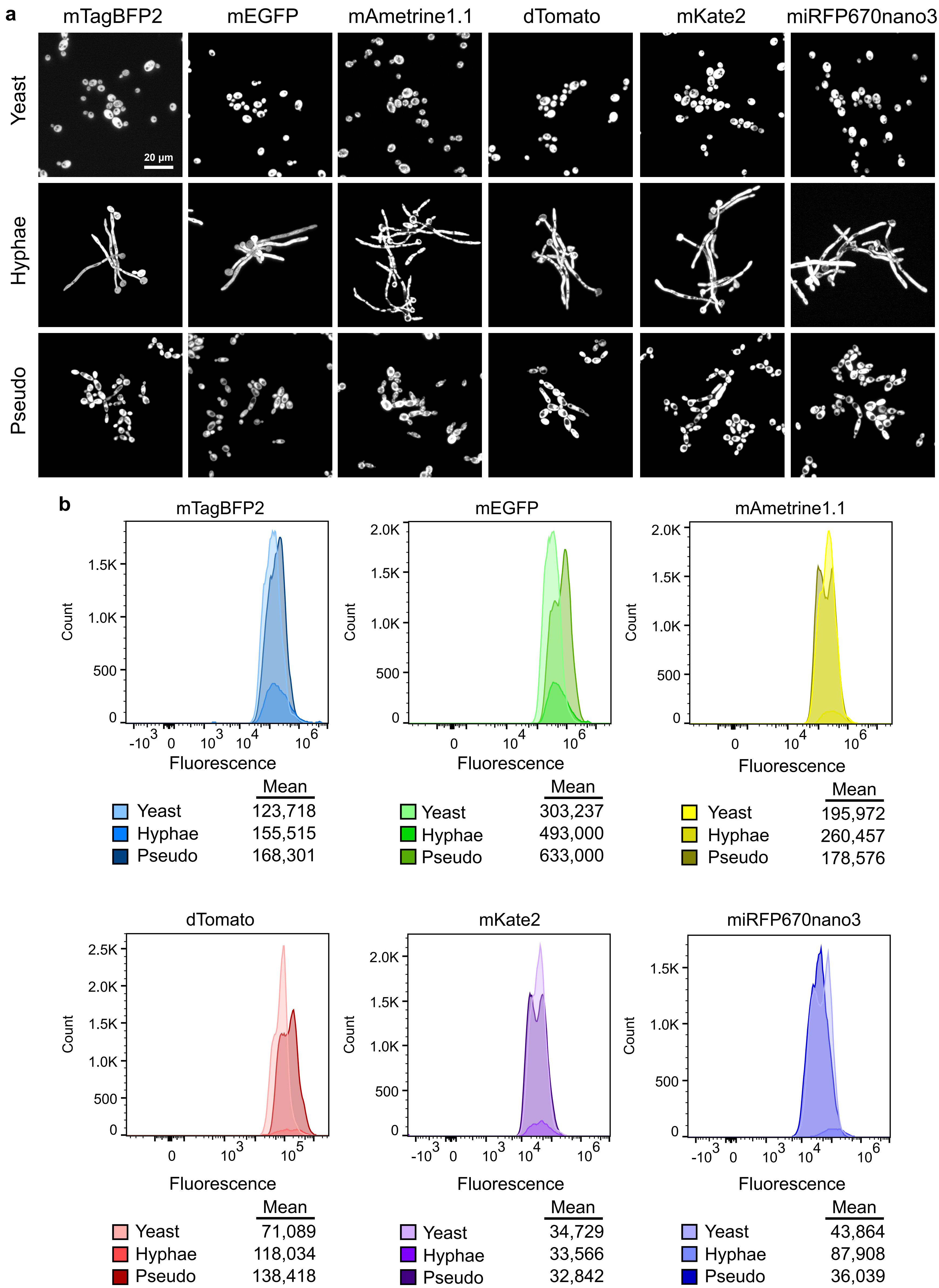

Figure S4. Fluorescence profiles of CandiChrome strains across yeast, hyphal, and pseudohyphal morphologies.
a, Representative fluorescence microscopy images of single-color CandiChrome strains expressing mTagBFP2, mEGFP, mAmetrine1.1, dTomato, mKate2, or miRFP670nano3 under yeast, hyphal, and pseudohyphal growth conditions. Yeast cells were grown overnight in SCD at 30 °C, hyphae were induced in RPMI supplemented with serum at 37 °C for 4 h, and pseudohyphae were induced in RPMI at 30 °C for 6 h. Scale bar, 20 µm. **b,** Representative flow cytometry fluorescence intensity distributions of the same fluorophore-expressing strains following classification into yeast, hyphal, and pseudohyphal populations. Mean fluorescence intensities are indicated for each morphological state.

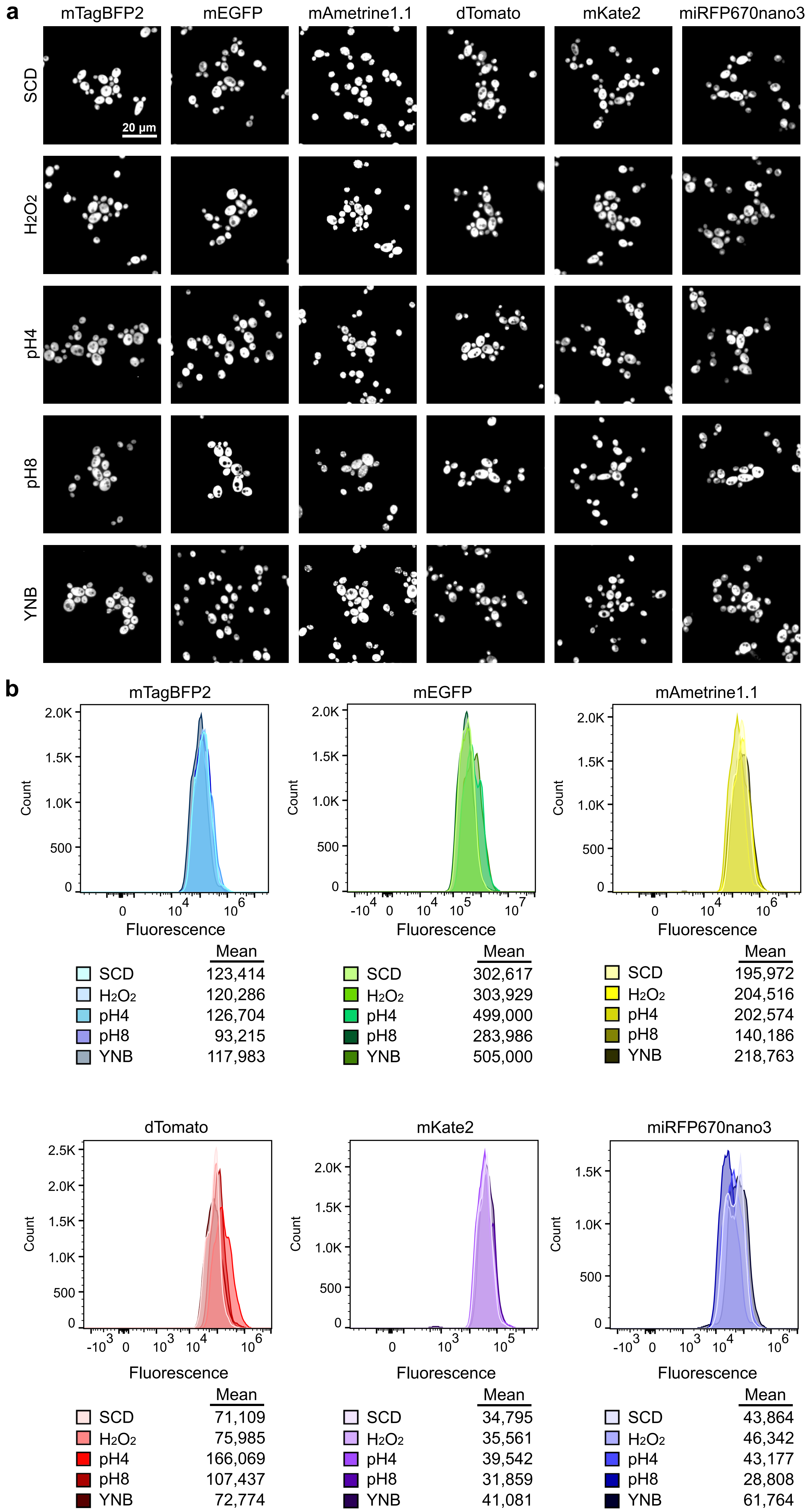

Figure S5. Fluorescence profiles of CandiChrome strains under oxidative, pH, and nutrient-limited stress conditions.

a, Representative fluorescence microscopy images of single-color CandiChrome strains expressing mTagBFP2, mEGFP, mAmetrine1.1, dTomato, mKate2, or miRFP670nano3 after exposure to SCD, oxidative stress, acidic or alkaline pH, or nutrient-limited conditions. Unless otherwise indicated, cells were grown overnight at 30 °C under the indicated conditions. For oxidative stress, cells were first grown overnight in SCD at 30 °C and then treated with 1 mM H_2O_2 for 1 h. Scale bar, 20 µm. **b,** Representative flow cytometry fluorescence intensity distributions of the same fluorophore-expressing strains under the corresponding conditions. Mean fluorescence intensities are indicated for each condition.

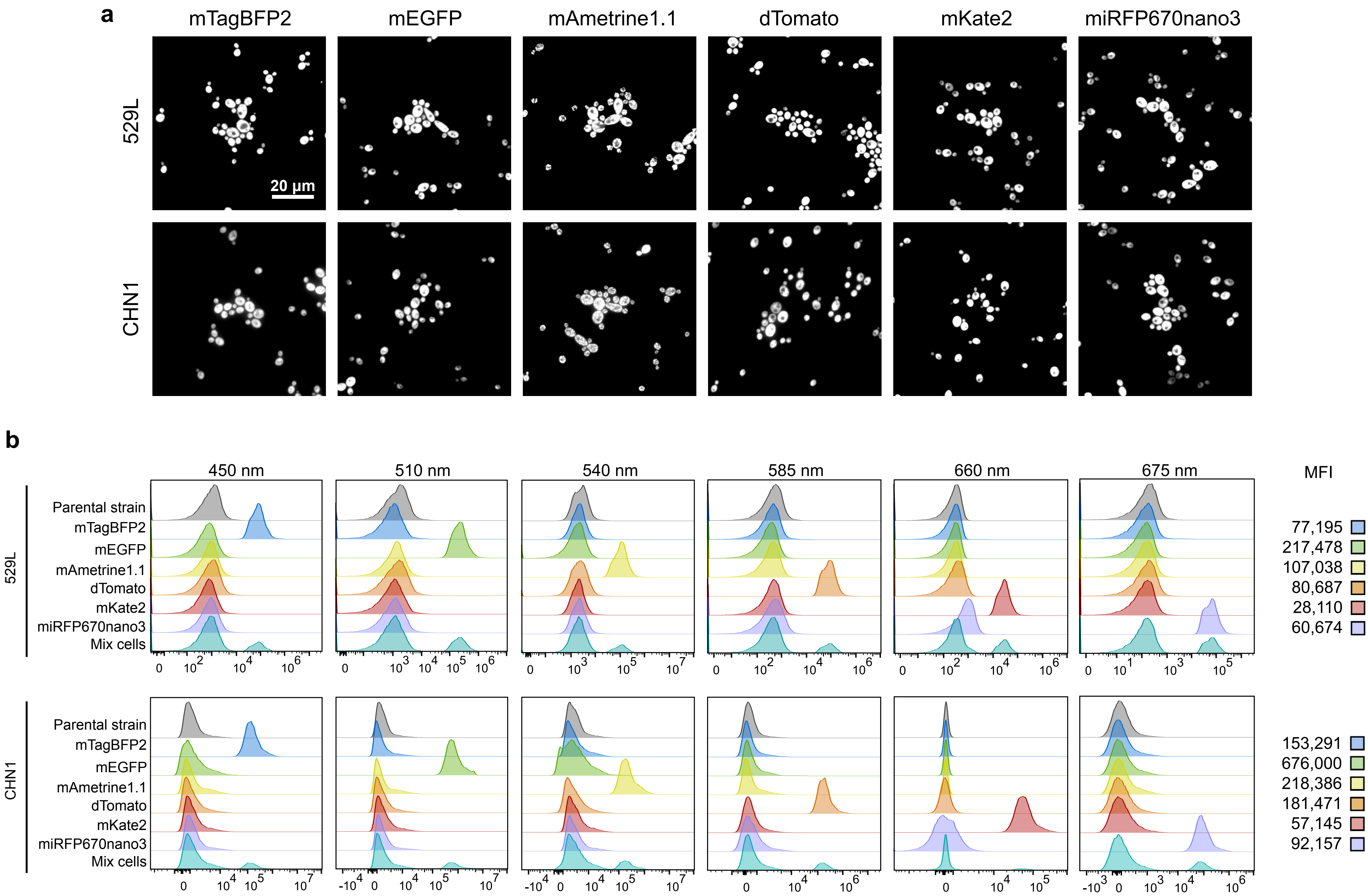

Figure S6. Validation of the CandiChrome platform in additional Candida albicans strain backgrounds.

a, Representative fluorescence microscopy images of single-color CandiChrome strains expressing mTagBFP2, mEGFP, mAmetrine1.1, dTomato, mKate2, or miRFP670nano3 in the 529L and CHN1 *C. albicans* strain backgrounds. Cells were grown overnight in SCD medium at 30°C. **b,** Representative flow cytometry fluorescence intensity distributions of parental, single-color, and mixed CandiChrome strains in the 529L and CHN1 backgrounds across the indicated fluorescence detection channels. Mean fluorescence intensity values are shown for each fluorophore-expressing strain.

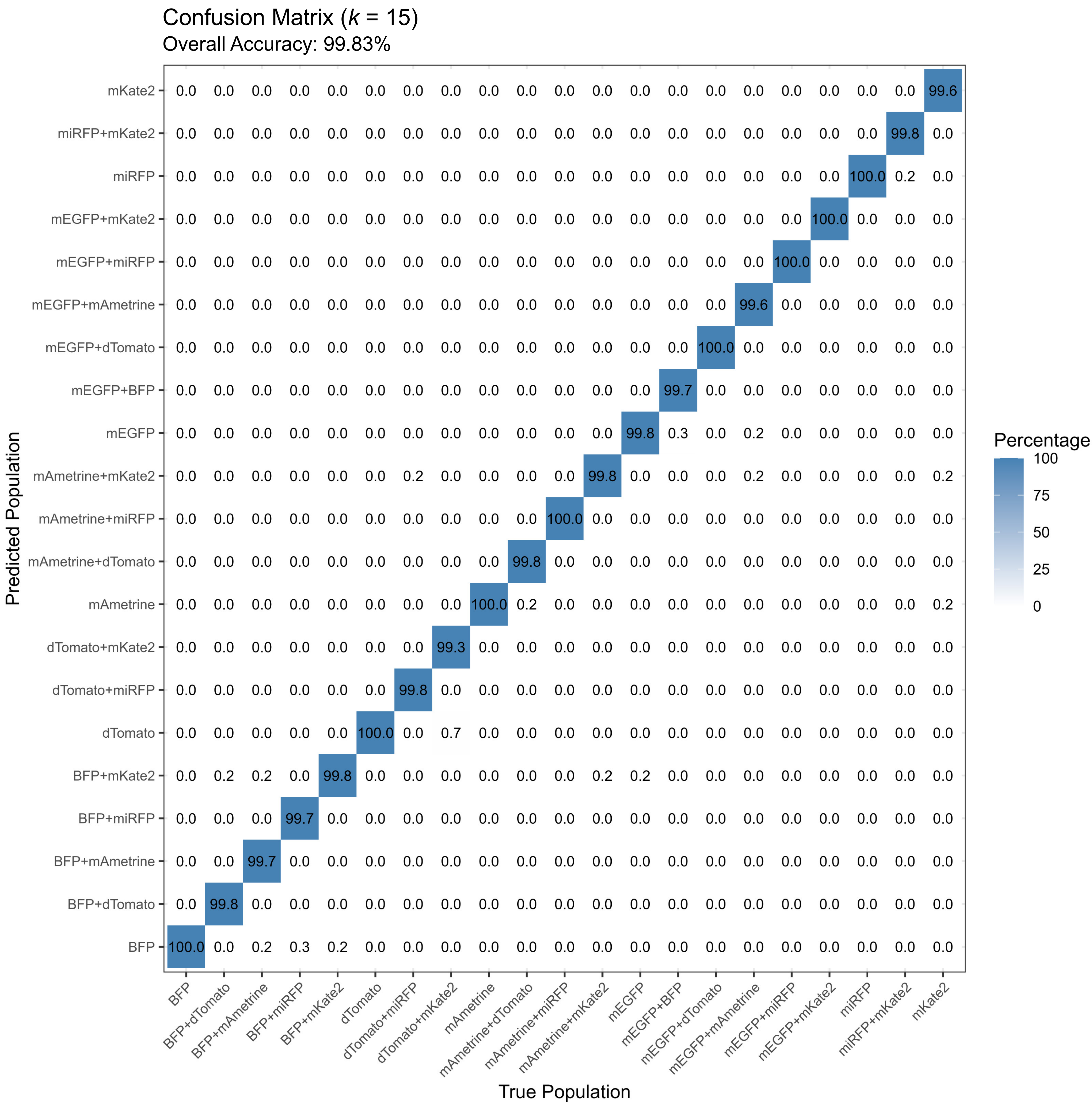

Figure S7. *k*-nearest-neighbor classification validation of 21 CandiChrome-labeled populations.

Confusion matrix showing classification performance of the *k*-nearest-neighbor classifier for the 21 single- and double-labeled *C. albicans* populations. Events from the known 21-population reference dataset were randomly split into a 70% training set and a 30% hold-out testing set. The training set was used for 5-fold cross-validation to optimize the *k* parameter, and *k* = 15 was selected for final classification. The optimized classifier was then applied to the independent testing set. Matrix values indicate the percentage of events from each true population assigned to each predicted population. Diagonal values indicate correctly classified events, while off-diagonal values indicate misclassified events. The overall classification accuracy was 99.83%.

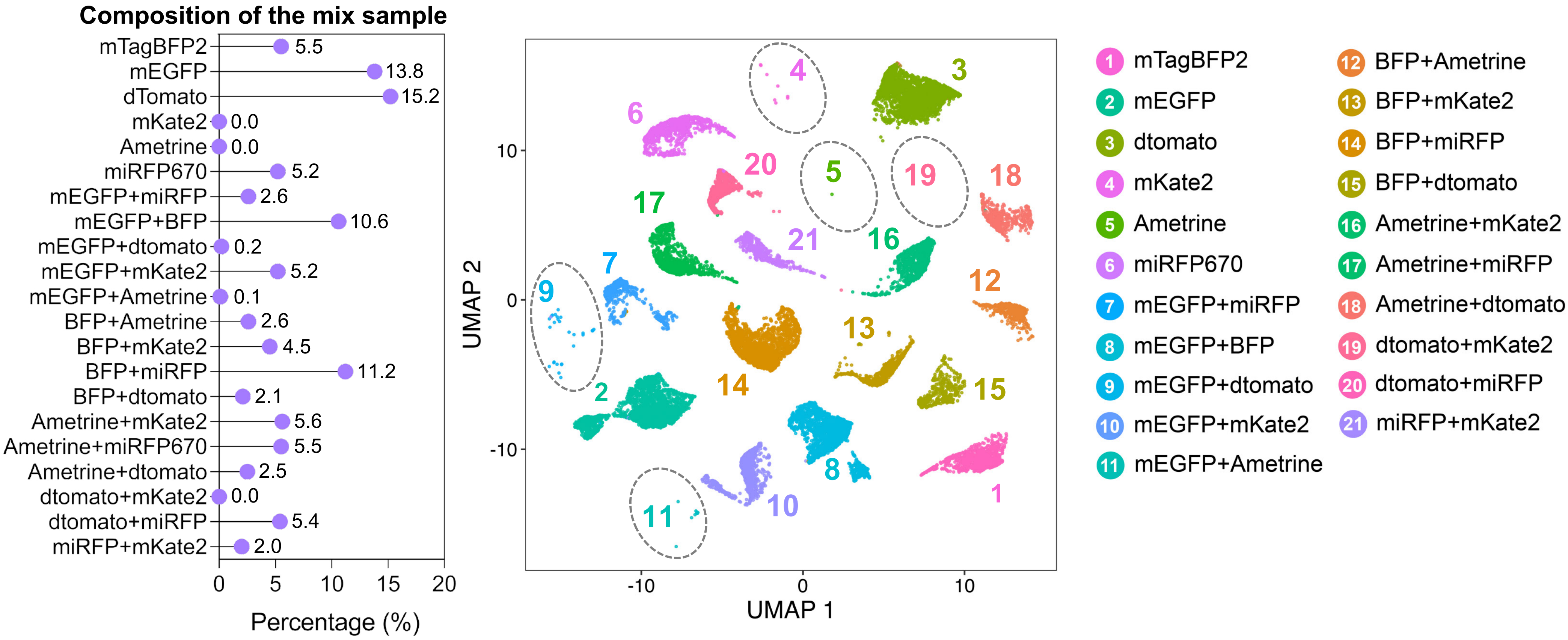

Figure S8. Resolution of a 16-population sample by flow cytometry and UMAP.
Composition of the mixed sample (left), UMAP projection (center), and population labels (right). Data were acquired on a Cytek Aurora flow cytometer and analyzed in R.

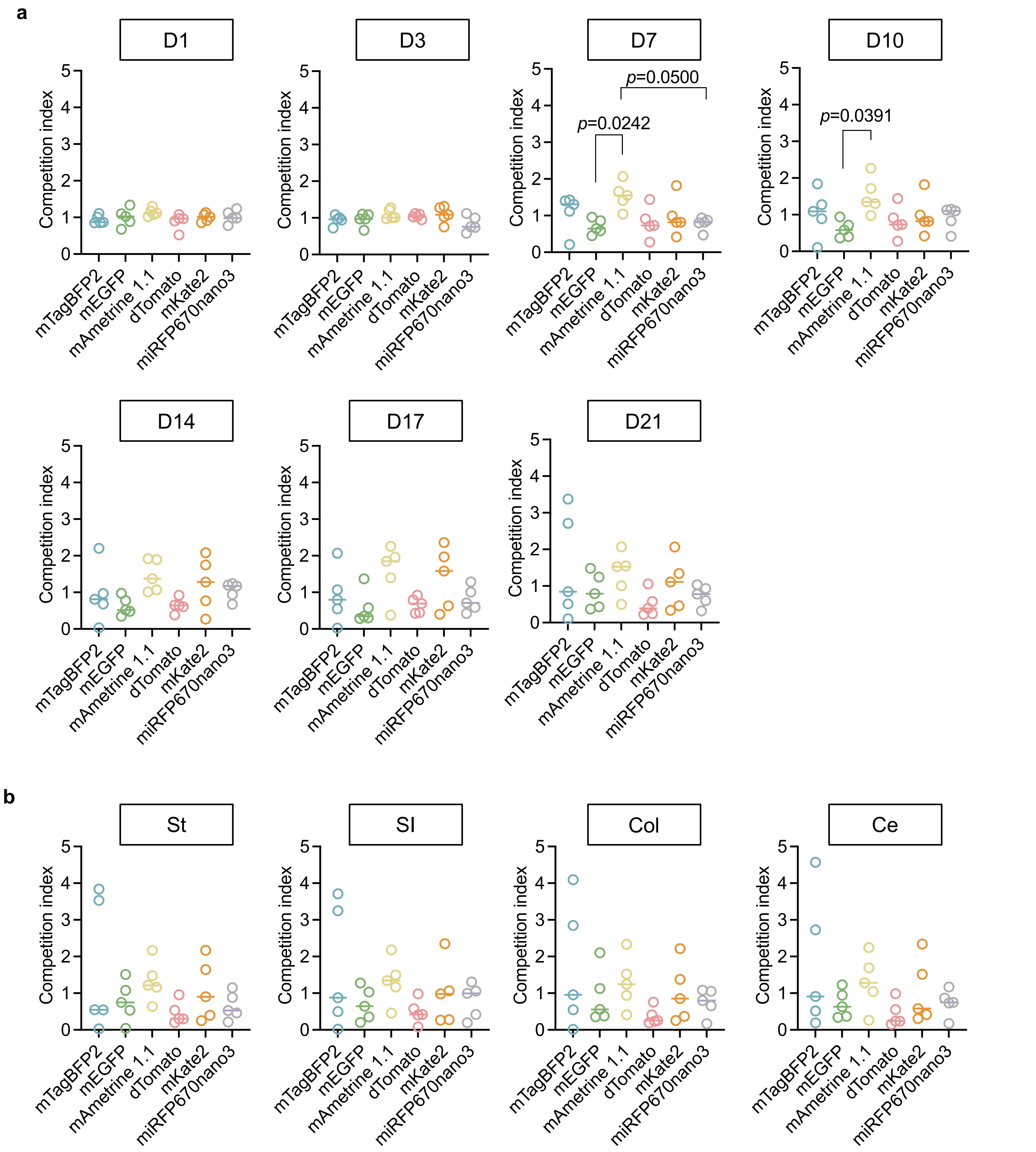

Figure S9. Competition indices of CandiChrome-labeled strains during gastrointestinal colonization.

a, Competition index values for each CandiChrome-labeled strain recovered from fecal samples at the indicated time points. Each symbol represents one mouse; horizontal lines indicate the mean. **b,** Competition indices for each strain in gastrointestinal tissues at endpoint. St, stomach; SI, small intestine; Col, colon; Ce, cecum. Each symbol represents one mouse; horizontal lines indicate the mean. Statistical significance was assessed by ordinary one-way ANOVA with Tukey's multiple-comparisons test. Exact P values are shown in the figure.

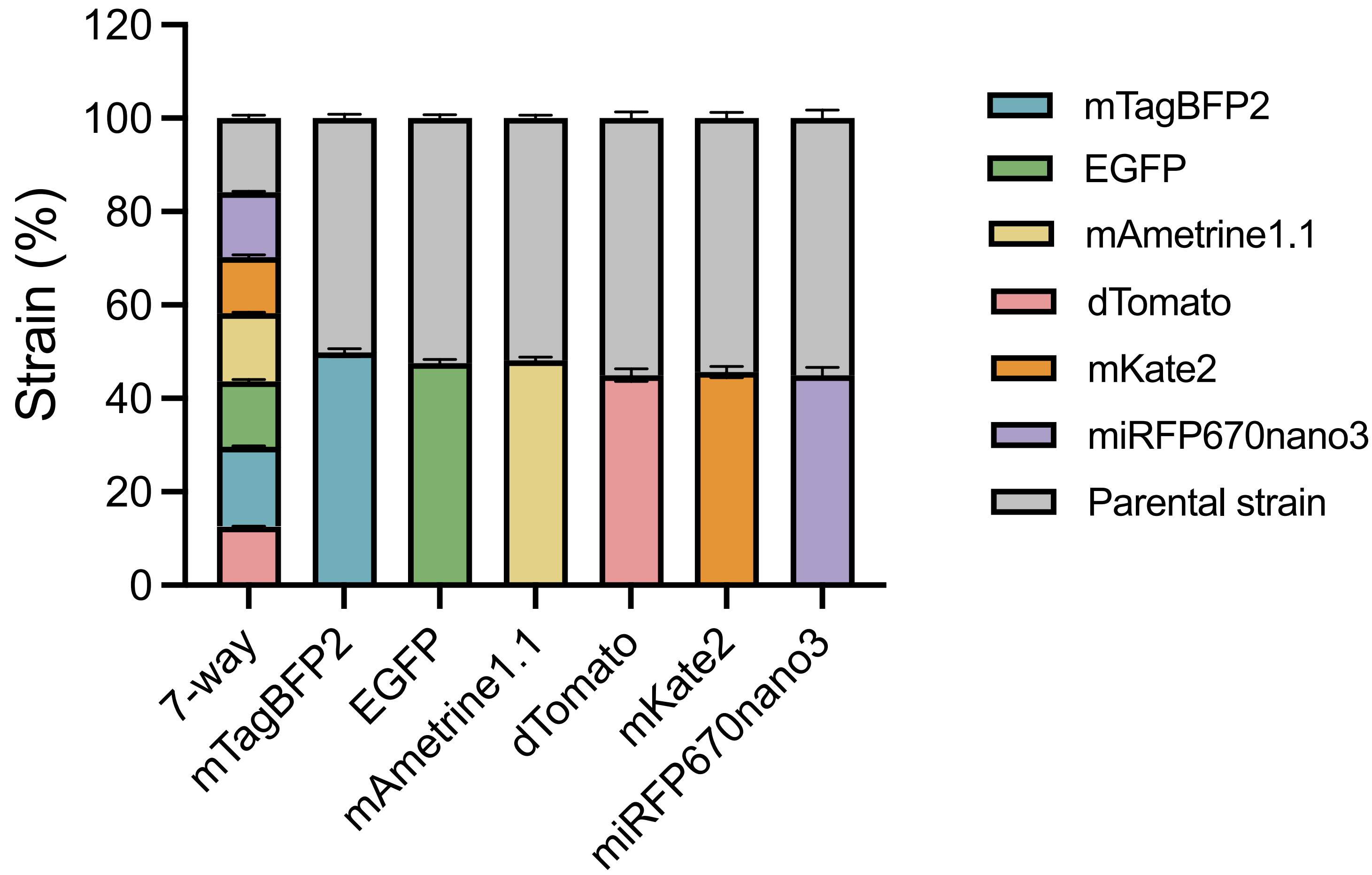

Figure S10. Direct *in vitro* competition assays between CandiChrome-labeled strains and the unlabeled parental control. A seven-way competition was performed by mixing the six flow-compatible CandiChrome strains expressing mTagBFP2, mEGFP, mAmetrine1.1, dTomato, mKate2, or miRFP670nano3 with the unlabeled parental strain. Pairwise 1:1 competitions were also performed between each fluorescently labeled strain and the unlabeled parental strain. Competitions were carried out in SCD medium at 30°C for 24 h, and strain proportions were quantified using an Aurora flow cytometer. Stacked bars indicate the percentage of each strain recovered after competition. Data are shown as mean ± s.d. from biological replicates.

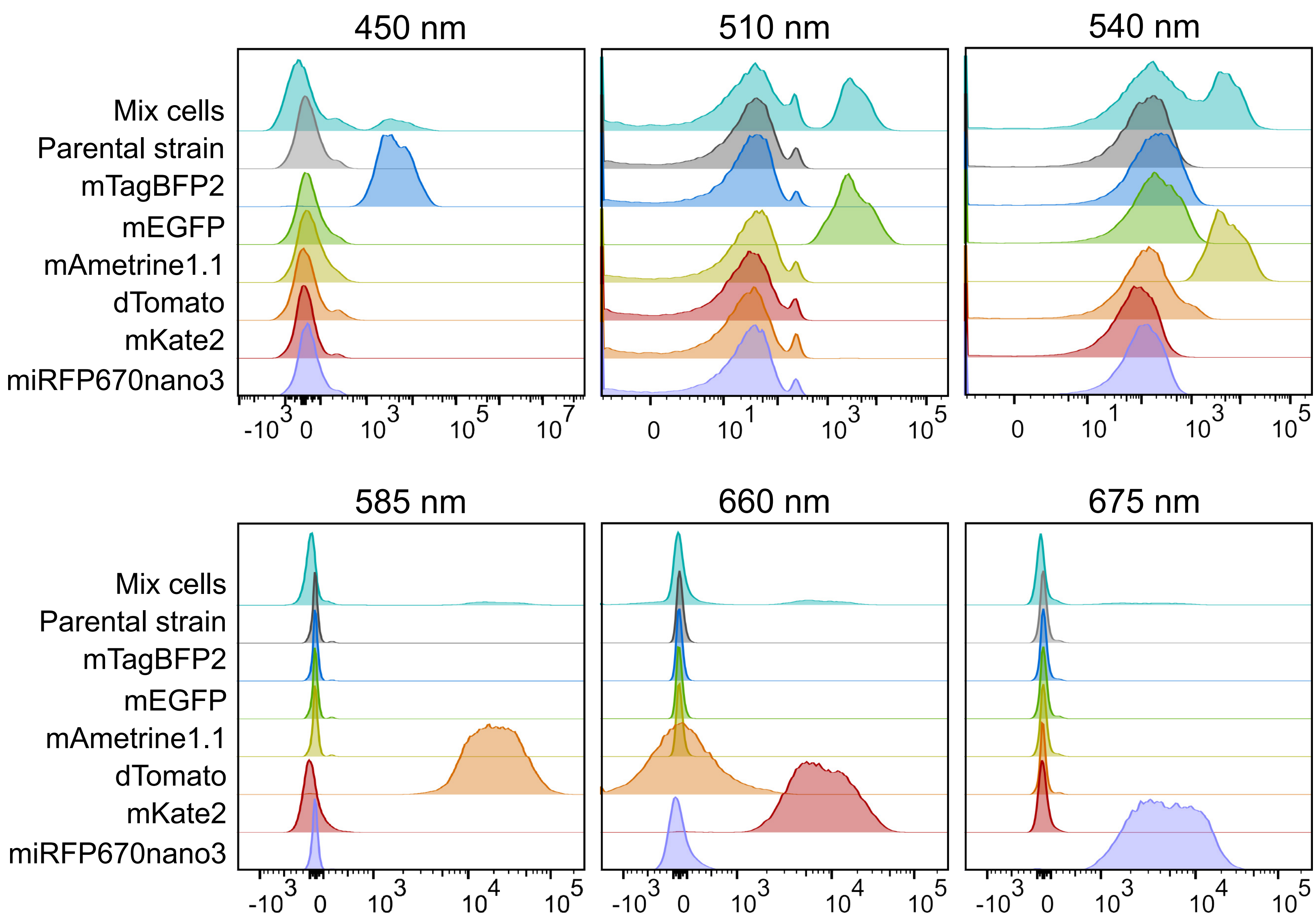

Figure S11. Representative flow cytometry profiles of an *in vivo*-derived CandiChrome sample.
Fluorescence intensity distributions in the indicated emission channels for a mixed-cell sample recovered from day 1, Comp5 of the *in vivo* competition assay. Individual CandiChrome strains and the parental strain (SC5314) are shown for comparison.

Table S1. Strains used in this study.

Strains	Genotype	Source
CAY12597	SC5314 (Wild-type)	[1]
CAY18965	529L	
CAY10076	CHN1	
CAY16975	SC5314 Eno1-mTagBFP2 fusion	This study
CAY17099	SC5314 Eno1- mTurquoise2 fusion	This study
CAY16965	SC5314 Eno1- mStayGold fusion	This study
CAY16971	SC5314 Eno1- mNeonGreen fusion	This study
CAY17002	SC5314 Eno1- mEGFP fusion	This study
CAY17060	SC5314 Eno1- mAmetrine1.1 fusion	This study
CAY17323	SC5314 Eno1- dTomato fusion	This study
CAY16999	SC5314 Eno1- mScarlet-I fusion	This study
CAY16902	SC5314 Eno1- mScarlet3-S2 fusion	This study
CAY17058	SC5314 Eno1- mKate2 fusion	This study
CAY17299	SC5314 Eno1- miRFP670nano3 fusion	This study
CAY17295	SC5314 Eno1- BDFP1.6 fusion	This study
CAY17302	SC5314 Eno1- smURFP fusion	This study
CAY17802	SC5314 pENO1- mTagBFP2 Neut5L	This study
CAY18103	SC5314 pENO1- mTurquoise2 Neut5L	This study
CAY18111	SC5314 pENO1- mStayGold Neut5L	This study
CAY17804	SC5314 pENO1- mNeonGreen Neut5L	This study
CAY18129	SC5314 pENO1- mEGFP Neut5L	This study
CAY18106	SC5314 pENO1- mAmetrine1.1 Neut5L	This study
CAY18109	SC5314 pENO1- dTomato Neut5L	This study
CAY18127	SC5314 pENO1- mScarlet-I Neut5L	This study
CAY18131	SC5314 pENO1- mScarlet3-S2 Neut5L	This study
CAY18114	SC5314 pENO1- mKate2 Neut5L	This study
CAY18116	SC5314 pENO1- miRFP670nano3 Neut5L	This study
CAY18126	SC5314 pENO1- BDFP1.6 Neut5L	This study
CAY18321	SC5314 pENO1- smURFP Neut5L	This study
CAY19263	SC5314 Cox4-dTomato; Vph1-miRFP670nano3; Efg1-mTagBFP2;Ras1-mNeonGreen	This study
CAY18410	SC5314 pENO1-mEGFP+ pENO1-miRFP670nano3 Neut5L	This study
CAY18333	SC5314 pENO1-mEGFP+ pENO1-mTagBFP2 Neut5L	This study

CAY18336	SC5314 pENO1-mEGFP+ pENO1-dTomato Neut5L	This study
CAY18335	SC5314 pENO1-mEGFP+ pENO1-mKate2 Neut5L	This study
CAY18334	SC5314 pENO1-mEGFP+ pENO1-mAmetrine1.1 Neut5L	This study
CAY18407	SC5314 pENO1-mTagBFP2+ pENO1-mAmetrine1.1 Neut5L	This study
CAY18342	SC5314 pENO1-mTagBFP2+ pENO1-mKate2 Neut5L	This study
CAY18344	SC5314 pENO1-mTagBFP2+ pENO1-miRFP670nano3 Neut5L	This study
CAY18405	SC5314 pENO1-mTagBFP2+ pENO1-dTomato Neut5L	This study
CAY18352	SC5314 pENO1-mAmetrine1.1 + pENO1-mKate2 Neut5L	This study
CAY18350	SC5314 pENO1-mAmetrine1.1 + pENO1-miRFP670nano3 Neut5L	This study
CAY18341	SC5314 pENO1-mAmetrine1.1 + pENO1-dTomato Neut5L	This study
CAY18404	SC5314 pENO1-dTomato + pENO1-mKate2 Neut5L	This study
CAY18409	SC5314 pENO1-dTomato + pENO1-miRFP670nano3 Neut5L	This study
CAY18338	SC5314 pENO1-miRFP670nano3 + pENO1-mKate2 Neut5L	This study
CAY19659	529L pENO1- mTagBFP2 Neut5L	This study
CAY19661	529L pENO1- mEGFP Neut5L	This study
CAY19663	529L pENO1- dTomato Neut5L	This study
CAY19667	529L pENO1- mAmetrine1.1 Neut5L	This study
CAY19669	529L pENO1- miRFP670nano3 Neut5L	This study
CAY19665	529L pENO1- mKate2 Neut5L	This study
CAY19671	CHN1 pENO1- mTagBFP2 Neut5L	This study
CAY19676	CHN1 pENO1- mEGFP Neut5L	This study
CAY19677	CHN1 pENO1- dTomato Neut5L	This study
CAY19679	CHN1 pENO1- mAmetrine1.1 Neut5L	This study
CAY19675	CHN1 pENO1- miRFP670nano3 Neut5L	This study
CAY19673	CHN1 pENO1- mKate2 Neut5L	This study

1. **Bennett RJ, Johnson AD.** 2006. The role of nutrient regulation and the Gpa2 protein in the mating pheromone response of *C. albicans*. Mol Microbiol **62:** 100-119.

Table S2. Oligonucleotides used in this study.

Number	Sequence (5'->3')
6060	GGACCGCCGCGGTAAACAAGTGGTATTCAAGCACAAT
6061	GGACCGGAGCTCCAGGAAGGACGATGAAGGA
4568	GGACCGCTCGAGCGGATCCCCGGGTTAATT<br>AACGGTATGGTTTCAAAAGGTGAAGAAGTT<br>ATTAAAG
4569	GGACCGCTCGAGTTATTTATACAATTCATC<br>CATACCATACAAAAACAA
4438	CTCAACCATAGCAATCATGG
9795	GGACCGGGTCTCTCAGTCATTTGTATCTTTAGTAGACATGATTGT
9728	GGACCGGGTCTCTCATTGTTGTAATATTCCTGAATTATCAAT
9799	GGACCGGGTCTCTTAAAGTAAAACCAGACTTTGATTTGATT
9800	GGACCGGGTCTCTAGTATGGTAATAGGAAGTCAAAAGAAAGA
9880	GGACCGGGTCTCTAATGGTTTCAAAAGGTGAAGAAGTTATTAAAG
9881	GGACCGGGTCTCTTTTATTTATACAATTCATCCATACCATACAAAAAC
9882	GGACCGGGTCTCTAATGGGTGGTAGTGGTATGGTTTC
9883	GGACCGGGTCTCTTTTAGTTCAATTTATGACCTAATTTTGATGG
9884	GGACCGGGTCTCTTTTATTTATACAATTCATCCATACCCATAACA
10055	GGACCGGGTCTCTTTTATGACATAGCTTTAATAATATAATCAAAATATGGA<br>G
10047	GGACCGGGTCTCTTTTACTATCTATGACCTAATTTTGATGGTAAATCA
10054	GGACCGGGTCTCTTTTAAATTTCAGTTTCTGAAATAACTCTAGC
10049	GGACCGGGTCTCTTTTATGATTGTTGAATAGCAATACCCA
10053	GGACCGGGTCTCTTTTATTATAAATGAGCTTCTAAAGTTTCAGATTG
10048	GGACCGGGTCTCTTTTACTATTTATATAATTCATCCATACCTGGAGTA
10050	GGACCGGGTCTCTTTTACTATTTATATAATTCATCCATACCTAAAGTAATA<br>CCA
10056	GGACCGGGTCTCTTTTACTATGAACCACCTGAACCAC
10051	GGACCGGGTCTCTTTTACTAATATAATTCATCCATACCACCTG
10052	GGACCGGGTCTCTTTTATTATTTATATAATTCATCCATACCTAAAGTAATA<br>CCA
9547	ATCTTGAGAATCGAAGAAGAATTAGGTTCTGAAGCTATCTACGCTGGTAA<br>AGATTTCCAAAAGGCTTCTCAATTGGGTGGTAGTGGTATGGTTTCTAAAG
9548	TTTGACTGCAGCTCAGTGATTAAGAGTAAAGATGGGTAAAAAATTATCAT<br>TTAATTAGTTCATATATTCAAGATGGCGGCCGCTCTAGAACTAGTGGATC

9739	AGAATCGAAGAAGAATTAGGTTCTGAAGCTATCTACGCTGGTAAAGATTT CCAAAAGGCTTCTCAATTGGGTGGTAGTGGTATGGTTTCAAAAGGTGAAG
7764	GTGATGATTTGACTGTCACTAACC
9319	GTGACTCCATCACCCAGTTT
7763	GATGCTTGGGTCCACTTCT
4906	ACTTTAATCCTTAGTCTTCTATTCTGAGAT
7832	ACGCAATTAATGTGAGTTAGC
4439	GCGAAAAAGTGGGCACTAAG
4907	CAAATTCATTGGAGCGATATCG
10243	AAAGTCTCTAGATGCTGGCAATGTGGTACTGTCTTGAAGGCCAAATACTT GGGTGAACCAGGAATGGCTCATCATGGTGGTAGTGGTATGGTTTCAAAAG
10244	TAGTAGAGTTCGCCAAAAGAGAACCATGAAATATATATAAAAAATAGAA TATAGCAATTGTTGGGGGTCTGTCGGGCGGCCGCTCTAGAACTAGTGGAT C
10245	CTTTGATTGGTCCTGGTGCT
5222	CTTCACCTTCAATTTCAAATTCATGAC
10246	ATGTCGAAATATTTTGAAGGTGGTGGTTCTGCTTTTGAACCATTTACTTTT AAAGGTTTATTAGACAGTGTTTTAGGTGGTAGTGGTATGGTTTCTAAAG
10247	ATGTTTAATGTTTAATGTTTATTATATATATTCAAATAAATATAATTCTTAA TTCTTTTTTATAAATTAATTGAGGCGGCCGCTCTAGAACTAGTGGATC
10248	TAATGCATTTGGTCCCACCG
4360	GTGGTTTCAGTGGCTACAAC
4446	CAAGGTTCAGTTCACCCTTCACCCCAACAACATCAAGCTAATCAATCAGC TAGCACTGTTGCCAAAGAAGAAAAGGGTGGTAGTGGTATGGTTTCTAAAG
8774	CTTTCCAATCATTTGTTAATGAAATATATGCTATAATCTAATTTGGAATTT ATGGCAGAAAGCAGAAGGTGATGTACACCGGCCGCTCTAGAACTAGT
5021	AGCGGTAATGGGAACAGTATA
5088	GGACCGGGTACCGCATAATCTCTTTAAACT
5089	ATTATCTTCTTCACCTTTAGAAACCATGGT
5090	ATGGTTTCTAAAGGTGAAGAAGATAAT
5091	TTTATACAATTCATCCATACCCATAACATCA
5092	TGATGTTATGGGTATGGATGAATTGTATAA
5115	GGACCGCTCGAGGTTAGTTTAAGATTACAA
5101	TTGTCTTGGGAATGTAAATTTAAATGAAAT
